# LL-37 inhibits osteosarcoma progression by suppressing SQLE-mediated cholesterol synthesis via the PTEN/AKT/mTOR pathway

**DOI:** 10.1016/j.jbo.2026.100779

**Published:** 2026-06-24

**Authors:** Fatai Lu, Zhijun Liu, Huasheng Jiang, Hong Zheng, Wenhui Su, Baosen Zhou

**Affiliations:** a Department of Clinical Epidemiology and Evidence-Based Medicine, The First Affiliated Hospital of China Medical University, Shenyang, China; b Department of Orthopaedics, Affiliated Hospital of Guangdong Medical University, Zhanjiang, PR China; c Department of Biochemistry and Molecular Biology, College of Life Science, China Medical University, Shenyang, China

**Keywords:** Osteosarcoma, LL-37, SQLE, Cholesterol metabolism, PTEN/AKT/mTOR pathway, Apoptosis, Pyroptosis

## Abstract

Osteosarcoma (OS) is an aggressive primary bone malignancy with poor prognosis for metastatic and recurrent cases, highlighting an urgent need for novel therapeutic strategies. The human cathelicidin peptide LL-37 exerts context-dependent anti-tumor effects, yet its functional role in OS remains largely undefined. This study aimed to explore the anti-OS activity and underlying mechanisms of LL-37. Methods: In vitro experiments were performed using OS cell lines and normal human bone marrow mesenchymal stem cells (hBMSCs) to assess cell viability, clonogenic survival, migration, invasion, cell cycle distribution, cell death, and cholesterol metabolism. Transcriptomic profiling, siRNA-mediated knockdown, plasmid overexpression, and rescue experiments were conducted to validate key signaling pathways. The in vivo therapeutic efficacy of LL-37 was evaluated using a 143B cell xenograft model. Results: LL-37 selectively inhibited the viability of OS cells with minimal toxicity to hBMSCs. It significantly suppressed clonogenic survival, migration, and invasion, induced S-phase cell cycle arrest, and triggered both mitochondrial apoptosis and caspase-1/GSDMD-dependent pyroptosis. Transcriptomic analysis identified cholesterol biosynthesis as a key pathway downregulated by LL-37, with the rate-limiting enzyme squalene epoxidase (SQLE) markedly reduced. Mechanistically, LL-37 upregulated the tumor suppressor PTEN, thereby inhibiting the AKT/mTOR pathway and suppressing SREBP2/SQLE-mediated cholesterol synthesis. Rescue experiments confirmed that SQLE inhibition was required for the pro-apoptotic effects of LL-37. In vivo, LL-37 dose-dependently inhibited the growth of OS xenografts with efficacy comparable to cisplatin, without causing obvious systemic toxicity. Conclusion: LL-37 exerts anti-OS effects by targeting the PTEN/AKT/mTOR-SREBP2/SQLE axis to suppress cholesterol synthesis and induce dual cell death. It represents a promising and selective candidate, providing a theoretical basis for cholesterol metabolism-targeted therapy in osteosarcoma.

## Highlights

• LL-37 inhibits proliferation and triggers apoptosis & pyroptosis in osteosarcoma cells, with low toxicity to normal human bone marrow mesenchymal stem cells.
• Mechanistically, LL-37 suppresses cholesterol synthase SQL via PTEN/AKT/mTOR axis to block cholesterol biosynthesis in osteosarcoma.
• Rescue experiments verify SQLE inhibition as the core mediator of LL-37’s anti-tumor activity against osteosarcoma.
• In vivo, LL-37 restrains osteosarcoma xenograft growth with cisplatin-comparable efficacy and negligible systemic toxicity.
• This work reveals a new crosstalk between LL-37, cholesterol metabolism and PTEN/AKT/mTOR, offering a therapeutic candidate for osteosarcoma.

## 1 Background

Osteosarcoma (OS) is the most common primary malignant bone tumor, mainly occurring in children, teenagers and young adults aged 10–25 years old, with a high incidence peak among those 10 to 20 years old [1], [2]. Despite advancements in multimodal therapy combining aggressive surgery with neoadjuvant, adjuvant, and multi-agent chemotherapy, as well as the emergence of targeted therapy, the clinical management of OS remains challenging [3], [4]. Over decades, the 5-year cumulative survival rate of OS patients has plateaued at around 60–70% with no notable improvement, while patients presenting with metastatic or recurrent disease experience a far gloomier clinical prognosis, whose 5-year survival stands at below 30% and in actual clinical treatment, even drops to 20% [5], [6]. This poor outcome is further compounded by multiple factors: the high degree of genomic heterogeneity of OS, characterized by complex karyotypes with abundant structural and numerical aberrations, which has historically hindered the discovery of driver genes and the development of targeted therapies [7], [8]; the limitations of first-line chemotherapeutic agents (cisplatin, doxorubicin, and methotrexate), whose efficacy is constrained by severe systemic toxicity and acquired drug resistance; and the limited responsiveness to immunotherapeutic strategies due to the immunosuppressive tumor microenvironment mediated by factors such as M2 macrophage polarization [9], [10]. Moreover, despite the common recurrence of alterations in tumor suppressors such as TP53 and RB1, the absence of a widely prevalent defining fusion product poses a significant hurdle to therapeutic advancement [11], [12]. Thus, there is an urgent and unmet need to identify novel, safe, and effective therapeutic agents with higher efficacy and selectivity against OS cells, as well as to elucidate their underlying molecular mechanisms, to improve the clinical outcomes of OS patients.

Human cathelicidin antimicrobial peptide LL-37, the only cationic antimicrobial peptide (CAMP) derived from human cathelicidin, is a key component of the innate immune system, renowned for its broad-spectrum antimicrobial activity against bacteria, fungi, and viruses [13], [14]. Accumulating mechanistic investigations have delineated multifaceted anticancer modes of LL-37, which can be classified into membranolytic and non-membranolytic regulatory patterns [15]. Structurally, the cationic amphipathic α-helix of LL-37 enables selective binding to negatively charged membrane components of tumor cells, triggering cell death via a carpet-like membrane disruption model. Beyond direct membranolytic damage, LL-37 modulates a series of core oncogenic signaling axes, including GPCR/p53/Bcl-2, Wnt/β-catenin, MAPK, NF-κB, and BMP/proteasome pathways, thereby inducing cell cycle arrest, initiating both caspase-dependent and caspase-independent mitochondrial apoptosis, and regulating AIF/EndoG-mediated cell death. Additionally, LL-37 remodels the tumor immune microenvironment by modulating immune cell infiltration and regulatory T cell activity, while its bioactive fragments exert anti-metastatic and anti-angiogenic effects in multiple solid tumors [16], [17]. Recent emerging studies further expanded the functional spectrum of LL-37, revealing its critical role in regulating lipid and cholesterol homeostasis by interacting with lipoprotein components and modulating intracellular lipid accumulation [18], [19]. Nevertheless, LL-37 displays context-dependent dual roles in tumor progression: it promotes proliferation and metastasis in breast, lung cancer and melanoma, whereas it exerts prominent tumor-suppressive effects in colorectal cancer and glioblastoma [16], [17], [20], [21]. Although LL-37 has been reported to regulate osteogenic differentiation in bone tissue engineering, its specific biological functions and detailed molecular mechanisms in OS remain largely unexplored [22]. Notably, osteosarcoma itself exhibits high genomic heterogeneity and a complex tumor microenvironment [23], which provides a context for the potential complex role of LL-37 therein. The known immunomodulatory functions of LL-37 [24] are closely associated with its potential mechanism of action in osteosarcoma, warranting further investigation and clarification.

Metabolic reprogramming, particularly of cholesterol biosynthesis, is a recognized hallmark of cancer, enabling tumor cells to meet the heightened demands for rapid proliferation, membrane biogenesis, and signal transduction [25], [26]. Squalene epoxidase (SQLE), a key rate-limiting enzyme in the cholesterol biosynthetic pathway that catalyzes the oxidation of squalene to 2,3-oxidosqualene, has emerged as a potential oncogene. Its aberrant upregulation is observed in multiple cancers (e.g., lymphomas，liver, lung and esophageal squamous cell carcinoma) and is closely associated with poor prognosis and chemoresistance [27], [28], [29], [30]. Accumulated evidence in recent years further confirms that SQLE is significantly overexpressed in OS and acts as a key oncogenic driver linking cholesterol metabolic reprogramming to OS deterioration, making it a promising therapeutic target [31], [32]. The transcription factor SREBP2 centrally controls the expression of cholesterol synthetic genes including SQLE. Meanwhile, the PTEN/AKT/mTOR pathway is frequently hyperactivated in OS and closely interplays with cholesterol metabolism, while the tumor suppressor PTEN functions as a key negative upstream regulator of this cascade [12], [33]. Although the individual roles of PTEN/AKT/mTOR, SREBP2/SQLE and cholesterol metabolism have been partially documented, the precise crosstalk among these axes in OS remains poorly clarified. More importantly, whether LL-37 inhibits OS progression by targeting the PTEN/AKT/mTOR-SREBP2/SQLE-mediated cholesterol metabolic network has never been reported.

Based on the aforementioned knowledge gaps, this study aimed to systematically investigate the anti-tumor effects of LL-37 on osteosarcoma (OS) and delineate its underlying molecular mechanisms. We first evaluated the selective cytotoxicity of LL-37 on OS cells versus normal bone marrow mesenchymal stem cells (hBMSCs), and subsequently examined its impact on OS cell clonogenicity, migration, invasion, cell cycle progression, and modes of cell death (apoptosis and pyroptosis). Transcriptomic profiling was employed to identify key pathways modulated by LL-37, with a focus on cholesterol biosynthesis. Through a combination of in vitro functional assays and an in vivo xenograft model, we validated the central regulatory role of the PTEN/AKT/mTOR-SREBP2/SQLE axis in mediating the anti-OS effects of LL-37. For the first time, we demonstrate that LL-37 upregulates PTEN expression, inhibits the PI3K/AKT/mTOR pathway, and subsequently suppresses the activation of SREBP2 and the expression of its downstream target SQLE, thereby disrupting cholesterol homeostasis. Concurrently, LL-37 triggers both mitochondrial apoptosis and caspase-1/gasdermin D (GSDMD)-mediated pyroptosis. These findings not only establish LL-37 as a promising therapeutic candidate for OS but also provide a novel theoretical foundation for OS treatment strategies that target cholesterol metabolism and induce dual cell death programs.

## 2 Methods

### 2.1 Cell lines and reagents

Human osteosarcoma (OS) cell lines U2OS, MG63, and 143B were purchased from Procell Life Science & Technology Co., Ltd. (Wuhan, China). Normal human bone marrow mesenchymal stem cells (hBMSCs) were obtained from Cyagen Biosciences (Guangzhou, China). All cells were authenticated by short tandem repeat (STR) profiling and tested negative for mycoplasma. U2OS, MG63, and 143B cells were cultured in high-glucose Dulbecco's Modified Eagle's Medium (DMEM, Gibco, Thermo Fisher Scientific) supplemented with 10% fetal bovine serum (FBS, Gibco) and 1% penicillin-streptomycin (Beyotime Biotechnology, Shanghai, China) at 37 °C in a 5% CO₂ humidified incubator. hBMSCs were cultured in OriCell® MSC Medium (Cyagen Biosciences, Guangzhou, China) per the manufacturer’s instructions.

Recombinant human antimicrobial peptide LL-37 (purity >98%) was purchased from GL Biochem (Shanghai, China), dissolved in sterile phosphate-buffered saline (PBS) to make a 10 mg/mL stock solution, and stored at −20 °C or − 80 °C. Cisplatin (positive control) was obtained from Sigma-Aldrich, and 2,3-oxidosqualene (2,3-OS) for rescue experiments from MedChemExpress. Primary antibodies for Western blotting and immunofluorescence—including PTEN, AKT, p-AKT (Ser473), mTOR, p-mTOR (Ser2448), SREBP2, SQLE, caspase-1, cleaved caspase-1, GSDMD, cleaved GSDMD, Bax, Bcl-2, and loading control β-actin—were all from Cell Signaling Technology (Danvers, MA, USA). Functional assay reagents (CCK-8, LDH release, Annexin V-FITC/PI apoptosis, TUNEL apoptosis, JC-1 mitochondrial membrane potential, ROS detection kits, and Filipin III for cholesterol staining) were purchased from Beyotime. Small interfering RNAs (si-PTEN and si-NC) and SQLE overexpression plasmid (SQLE-OE) with empty vector control were synthesized/constructed by GenScript (Nanjing, China). Cell transfection was performed using Lipofectamine 3000 (Invitrogen). Whole blood was collected from pathogen-free BALB/c nude mice for hemolysis assay.

### 2.2 Cell viability and cytotoxicity assays

Cell viability was evaluated via the CCK-8 assay (Beyotime) according to the manufacturer's instructions. U2OS, MG63, and hBMSCs were seeded into 96-well plates at 3 × 10^3^ cells/well and cultured overnight, then treated with LL-37 at gradient concentrations (0, 20, 40, 80 μg/mL for OS cells; 0, 80, 160, 320, 640 μg/mL for hBMSCs) for 3, 6, 12, 24, and 48 h. After treatment, 10 μL of CCK-8 solution was added to each well, followed by incubation at 37 °C for 2 h. The absorbance at 450 nm was measured using a microplate reader (Bio-Rad, Hercules, CA, USA). Cell viability was calculated as the percentage relative to the untreated control group, and half-maximal inhibitory concentration (IC₅₀) values were computed using GraphPad Prism 9.0 software (GraphPad Software, San Diego, CA, USA).

Cytotoxicity was assessed by measuring lactate dehydrogenase (LDH) release into the culture supernatant using an LDH Cytotoxicity Assay Kit (Beyotime). Cells were seeded into 96-well plates at 5 × 10^3^ cells/well and cultured overnight, then treated with LL-37 (0, 20, 40, 80 μg/mL for OS cells; 0, 80, 160, 320, 640 μg/mL for hBMSCs) for 24 h. Per the kit instructions, 120 μL of supernatant from each well was transferred to a new 96-well plate, mixed with 60 μL of LDH reaction mixture, and incubated at room temperature in the dark for 30 min. The absorbance at 490 nm was measured using a microplate reader, and the LDH release rate was calculated as the percentage relative to the maximum release group (cells treated with 1% Triton X-100).

Hemolytic activity of LL-37 was evaluated using whole blood collected from BALB/c nude mice. All animal-related procedures were performed in accordance with the approved animal ethics guidelines. The mouse whole blood samples were incubated with serially diluted LL-37 at 37 °C for 24 h. After centrifugation, the absorbance of the supernatant was measured at 540 nm using a spectrophotometer to calculate the hemolysis rate.

### 2.3 Clonogenic, migration, and invasion assays

Clonogenic survival assay was used to assess long-term proliferative survival capacity of LL-37-treated (0, 20, 40,80 μg/mL) U2OS and MG63 cells; cells were seeded in 6-well plates at 500 cells/well, cultured overnight, and after 24-h LL-37 treatment, medium was replaced with fresh complete medium for another 10–14 days of colony formation. Colonies were fixed with 4% paraformaldehyde for 15 min, stained with 0.1% crystal violet at room temperature for 30 min, rinsed with water to remove excess stain, air-dried, and those with >50 cells were counted via ImageJ software (NIH, Bethesda, MD, USA), with colony formation rate expressed as a percentage relative to the control group.

Wound healing and Transwell assays (with/without Matrigel coating) were used to assess migratory and invasive capacities of LL-37-treated (0, 20, 40 μg/mL) U2OS and MG63 cells; for the wound healing assay, cells seeded in 6-well plates were cultured to confluence, a uniform wound was made with a sterile 200 μL pipette tip, debris was rinsed with PBS, serum-free DMEM containing LL-37 was added, wound images were captured at 0 and 24 h via an Olympus inverted microscope, and wound closure rate was calculated by ImageJ using the formula: (initial wound width − 24-h width)/initial width × 100%; for the Transwell assay, 8 μm pore Corning chambers were applied, with migration assays uncoated and invasion assays pre-coated with 50 μL 1:8-diluted Matrigel (37 °C, 4 h for gel formation), 5 × 10^4^ cells suspended in LL-37-containing serum-free DMEM were seeded into the upper chamber, while 600 μL 10% FBS-supplemented DMEM was added to the lower chamber as chemoattractant, after 24 h (migration) or 48 h (invasion) incubation at 37 °C, non-migrated/invaded cells on the upper membrane were removed with a cotton swab, cells on the lower surface were fixed with 4% paraformaldehyde and stained with 0.1% crystal violet, and counted in five random fields under an inverted microscope.

### 2.4 Cell cycle, apoptosis, and pyroptosis analysis

The effects of LL-37 on the cell cycle distribution, apoptosis, and pyroptosis of osteosarcoma cells were evaluated using flow cytometry, fluorescence microscopy, and Western blotting. Cell cycle analysis was performed using propidium iodide (PI) staining. U2OS and MG63 cells were seeded in 6-well plates at a density of 2 × 10^5^ cells per well. After overnight attachment, cells were treated with various concentrations of LL-37 for 24 h. The DNA content was analyzed using a flow cytometer (BD FACSCanto II), and the percentage of cells in G0/G1, S, and G2/M phases was determined using ModFit LT software. Apoptosis was detected via the Annexin V-FITC/PI double-staining assay. After LL-37 treatment, cells were harvested, resuspended in binding buffer, and stained with Annexin V-FITC and PI for 15 min in the dark at room temperature. The stained cells were immediately analyzed by flow cytometry to distinguish viable (Annexin V^−^/PI^−^), early apoptotic (Annexin V^+^/PI^−^), and late apoptotic/necrotic (Annexin V^+^/PI^+^) cell populations. Apoptosis was further confirmed by the TUNEL assay. Fixed and permeabilized cells were incubated with the TUNEL reaction mixture at 37 °C for 1 h. Nuclei were counterstained with DAPI, and TUNEL-positive cells (green fluorescence) were observed and counted under a fluorescence microscope. Pyroptosis was evaluated by detecting the cleavage of key executioner proteins, caspase-1 and GSDMD, using Western blot analysis. Additionally, the release of interleukin-1β (IL-1β) into the cell culture supernatant was measured using a commercial ELISA kit to further corroborate pyroptosis induction.

### 2.5 Mitochondrial membrane potential (ΔΨₘ) and ROS detection

Mitochondrial membrane potential (ΔΨₘ) was determined using the JC-1 assay kit (Beyotime Biotechnology). Cells were treated with LL-37 for 24 h, then incubated with JC-1 working solution at 37 °C for 20 min and rinsed with JC-1 buffer. Fluorescent images were captured by a fluorescence microscope, and the ratio of red (aggregated JC-1) to green (monomeric JC-1) fluorescence intensity was calculated via ImageJ software. Flow cytometry was further employed to quantify ΔΨₘ by measuring JC-1 fluorescence intensity. Intracellular ROS levels were detected using the fluorescent probe DCFH-DA (Beyotime Biotechnology). After 24-h LL-37 treatment, cells were incubated with DCFH-DA at 37 °C for 20 min, washed with PBS, and analyzed by flow cytometry to calculate the mean fluorescence intensity (MFI).

### 2.6 Western blot analysis

Total proteins were extracted from harvested cells using RIPA lysis buffer (Beyotime) supplemented with protease and phosphatase inhibitors. The concentration of total protein was determined by the BCA protein assay kit (Beyotime). Equal amounts of protein (30 μg per lane) were separated by SDS-PAGE and then transferred onto PVDF membranes (Millipore, Burlington, MA, USA). The membranes were blocked with 5% non-fat milk in TBST for 1 h at room temperature, followed by incubation with primary antibodies overnight at 4 °C. After washing with TBST, the membranes were incubated with horseradish peroxidase (HRP)-conjugated secondary antibodies for 1 h at room temperature. The primary antibodies included those against SREBP2, SQLE, PTEN, AKT, p-AKT, mTOR, p-mTOR, caspase-1, GSDMD, Bcl-2, Bax, Cleaved Caspase-3/9, IL-1β, IL-18, and β-actin (loading control), all purchased from Cell Signaling Technology. Protein bands were visualized using an enhanced chemiluminescence (ECL) detection kit (Thermo Fisher), and the band intensity was quantified by ImageJ software with β-actin as the internal control.

### 2.7 Intracellular cholesterol detection

Intracellular free cholesterol (FC) and total cholesterol (TC) levels were quantified using cholesterol assay kits following the manufacturer's protocols. Cells were treated with LL-37for 24 h, then harvested, lysed, and centrifuged. The supernatant was used for FC and TC detection, and the levels were normalized to total protein concentration. Intracellular free cholesterol was visualized by Filipin III staining: fixed cells were stained with Filipin III at 4 °C in the dark for 2 h, observed under a fluorescence microscope, and the fluorescence intensity was quantified using ImageJ software.

### 2.8 Transcriptomic profiling and functional validation assays

To investigate the global gene expression profile changes in osteosarcoma cells induced by LL-37, transcriptomic sequencing and analysis were performed. U2OS cells were treated with 40 μg/mL LL-37 or PBS (control) for 24 h, and total RNA was extracted using TRIzol reagent. RNA quality was assessed using a NanoDrop 2000 spectrophotometer and an Agilent 2100 Bioanalyzer (RIN ≥ 7, 28S/18S ratio ≥ 1.5). Qualified RNA samples were subjected to paired-end sequencing on the Illumina NovaSeq 6000 platform by BerryGenomics Technology Co., Ltd. (Beijing, China). After quality control and filtering of raw sequencing data, differential expression analysis was performed using the DESeq2 R package (v1.28.0), with genes meeting the criteria of |log₂ fold change (FC)| > 1 and adjusted *P*-value <0.05 considered differentially expressed. Volcano plots and heatmaps were generated using R software (v4.0.2) for visualization. Gene Ontology (GO) enrichment analysis and Kyoto Encyclopedia of Genes and Genomes (KEGG) pathway analysis were conducted on the differentially expressed genes using the ClusterProfiler R package to identify significantly enriched biological processes and signaling pathways. Furthermore, public databases including UALCAN, Kaplan-Meier Plotter, and GEO datasets (GSE42352, GSE16089, GSE99671) were mined to analyze SQLE expression levels in osteosarcoma and their association with patient survival outcome.

Functional Validation Experiments (RNA Interference, Plasmid Transfection, and Rescue Experiments): To validate the necessity of PTEN in LL-37's effects, U2OS and MG63 cells were seeded in 6-well plates at a density of 2 × 10^5^ cells per well and cultured until 50–60% confluence. Cells were transfected with PTEN-targeting small interfering RNA or negative control siRNA using Lipofectamine 3000 according to the manufacturer's instructions. After 48 h of transfection, cells were treated with LL-37 (80 μg/mL) for 24 h, and protein expression of PTEN, p-AKT, p-mTOR, and SQLE was detected by Western blot. To validate the crucial role of SQLE, cells were transfected with an SQLE overexpression plasmid or an empty vector control using Lipofectamine 3000. After 48 h of transfection, cells were similarly treated with LL-37. Rescue experiments were performed in two ways: 1) co-treatment of cells with LL-37 and the metabolic product of SQLE; and 2) treatment of SQLE-overexpressing cells with LL-37. Following these treatments, intracellular cholesterol levels and apoptosis rates were re-evaluated using the methods described in previous sections to confirm the causal link between LL-37-induced inhibition of the SREBP2/SQLE axis, subsequent reduction in cholesterol synthesis, and induction of cell death.

### 2.9 In vivo xenograft model

All animal procedures were approved by the Institutional Animal Care and Use Committee. Female BALB/c nude mice (4–6 weeks old) were purchased from Beijing Vital River Laboratory Animal Technology Co., Ltd. (Beijing, China) and housed under specific pathogen-free (SPF) conditions. Xenograft models were established by subcutaneously injecting 143B cells (5 × 10^6^ cells in 100 μL of PBS mixed with Matrigel at a 1:1 ratio) into the right flank of each mouse. When the tumor volume reached approximately 100 mm^3^, the mice were randomly divided into four groups (*n* = 3 per group): control group (PBS), low-dose LL-37 group (5 mg/kg), high-dose LL-37 group (10 mg/kg), and cisplatin group (5 mg/kg, positive control). LL-37 and cisplatin were administered intraperitoneally every other day for 2 weeks, and mouse body weights as well as tumor volumes (calculated by the formula: V = (length × width^2^)/2) were recorded every 2 days. At the end of the experiment, the mice were sacrificed, and the tumors were collected, excised, weighed, fixed in 4% (*v*/v) paraformaldehyde solution, embedded in paraffin, and sectioned into 5 μm slices; the sections were then stained with hematoxylin and eosin (H&E), Ki67 antibody, and TUNEL kits, respectively, for histological analysis.

### 2.10 Statistical analysis

All experiments were independently repeated at least three times. Data are presented as the mean ± standard deviation (SD). Statistical analyses were performed using GraphPad Prism 9.0 software. Differences between two groups were analyzed by Student's *t*-test. Comparisons among multiple groups were conducted using one-way or two-way analysis of variance (ANOVA), followed by Tukey's post-hoc test for multiple comparisons. A *p*-value of less than 0.05 was considered statistically significant.

## 3 Results

### 3.1 LL-37 selectively inhibits osteosarcoma cell viability with minimal effects on normal cells

**Fig. 1:**
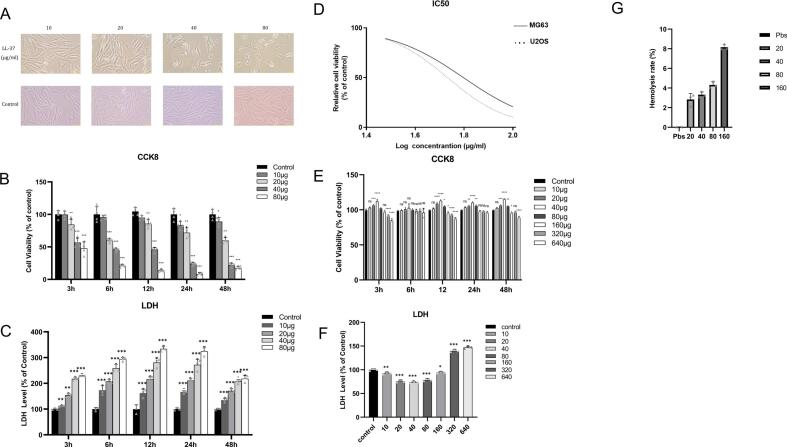
LL-37 selectively inhibits viability of osteosarcoma cells with minimal toxicity to normal hBMSCs. (A) Morphological changes of U2OS cells after treatment with LL-37. (B) Viability of U2OS cells treated with gradient concentrations of LL-37 for 3–48 h, detected by CCK-8 assay (*n* = 4 independent experiments). (C) LDH release in U2OS cells following LL-37 treatment, expressed as percentage of the control group (set as 100%) (*n* = 3). (D) IC50 values of LL-37 in U2OS and MG63 cells at 24 h (n = 3). (E) Viability of hBMSCs treated with high concentrations of LL-37 (n = 3). (F) LDH release in hBMSCs after LL-37 treatment, expressed as percentage of the control group (set as 100%) (n = 3). (G) Hemolysis assay was performed using whole blood from BALB/c nude mice with 24 h incubation at 37 °C (n = 3). *p < 0.05, **p < 0.01, ***p < 0.001 vs. control group.

To investigate the anti-tumor potential of LL-37, we first examined its effects on the viability of osteosarcoma (OS) cells. Microscopic observation of U2OS cells revealed that LL-37 treatment induced visible morphological changes and a reduction in cell density in a concentration-dependent manner (Fig. 1A). Quantitative assessment using a CCK-8 assay demonstrated that LL-37 (10–80 μg/mL) potently suppressed the viability of U2OS cells in a concentration- and time-dependent manner, with significant inhibition observed from 3 to 48 h (Fig. 1B). This cytotoxic effect was corroborated by a concurrent increase in lactate dehydrogenase (LDH) release, indicating loss of membrane integrity (Fig. 1C). Dose-response analysis yielded half-maximal inhibitory concentration (IC50) values of 54.01 μg/mL for U2OS cells and 62.77 μg/mL for MG63 cells after 24-h treatment (Fig. 1D), suggesting U2OS cells were slightly more sensitive. Importantly, LL-37 exhibited minimal cytotoxicity against normal human bone marrow mesenchymal stem cells (hBMSCs) at concentrations effective against OS cells, as no significant reduction in cell viability (Fig. 1E) or increase in LDH release (Fig. 1F) was observed even at much higher concentrations (up to 640 μg/mL). Together, these findings demonstrate that LL-37 selectively targets OS cells by significantly reducing their viability while sparing normal hBMSCs.

To further evaluate the biosafety profile of LL-37, we performed a hemolysis assay using whole blood collected from BALB/c nude mice with a 24 h incubation period. As shown in Fig. 1G, LL-37 induced only mild hemolysis in a dose-dependent manner. The hemolysis rates remained below 5% at concentrations up to 80 μg/mL, and reached approximately 8.2% at the highest tested concentration of 160 μg/mL. These data confirm that LL-37 exerts minimal hemolytic activity at the concentrations used in our in vitro and in vivo experiments.

Together, these findings demonstrate that LL-37 selectively targets OS cells by significantly reducing their viability while sparing normal hBMSCs and exhibiting low hemolytic potential.

### 3.2 LL-37 inhibits clonogenicity, motility, and induces cell cycle arrest in osteosarcoma cells

**Fig. 2:**
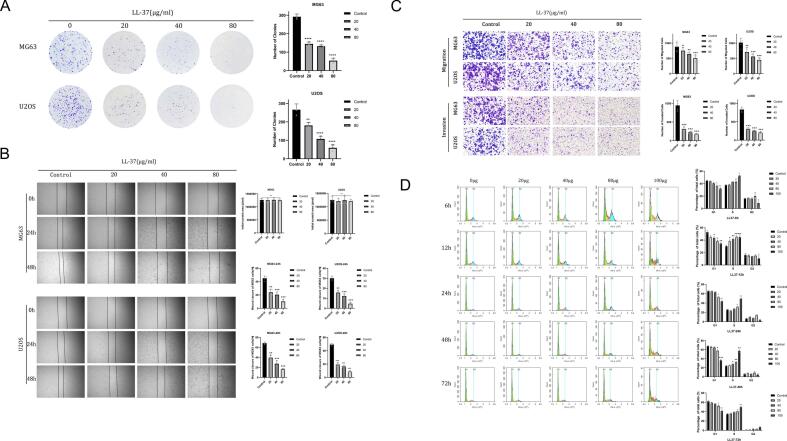
LL-37 suppresses clonogenicity, migration, invasion, and induces S-phase arrest in osteosarcoma cells. (A) Colony formation ability of U2OS and MG63 cells treated with LL-37 (n = 3). (B) Wound healing assay showing migration inhibition of osteosarcoma cells by LL-37 (n = 4). (C) Transwell migration and invasion assays of U2OS and MG63 cells after LL-37 treatment (n = 4). (D) Cell cycle distribution analyzed by flow cytometry, indicating S-phase arrest induced by LL-37 (n = 3). *p < 0.05, **p < 0.01, ***p < 0.001 vs. control group.

To investigate the anti-tumor potential of LL-37, we assessed its impact on key oncogenic properties of human osteosarcoma (OS) cell lines. The compound profoundly inhibited the long-term clonogenic survival of U2OS and MG63 cells in a concentration-dependent manner (Fig. 2A). Furthermore, LL-37 significantly impaired OS cell motility, as evidenced by dose-dependent inhibition in both wound healing (Fig. 2B) and Transwell migration/invasion assays (Fig. 2C). To explore the underlying mechanism for this growth suppression, we analyzed cell cycle progression. Flow cytometry revealed that LL-37 treatment induced a significant S-phase arrest, marked by an accumulation of cells in S-phase and a corresponding decrease in the G1-phase population (Fig. 2D). Together, these findings demonstrate that LL-37 effectively suppresses the clonogenicity and metastatic behaviors of OS cells, an effect potentially linked to its induction of S-phase cell cycle arrest.

### 3.3 LL-37 induces apoptosis and pyroptosis in osteosarcoma cells via mitochondrial dysfunction

**Fig. 3:**
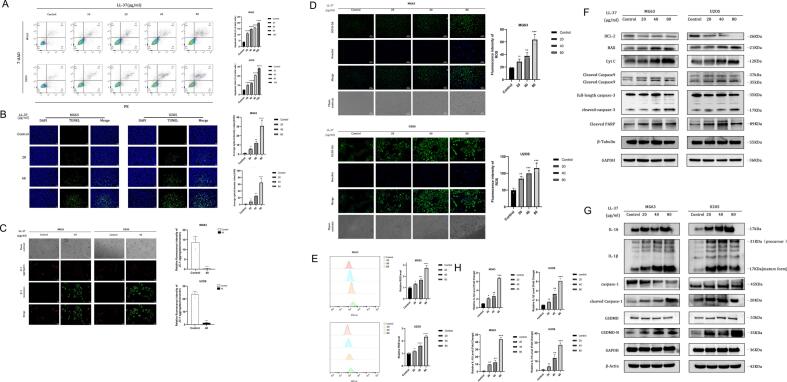
LL-37 triggers mitochondrial apoptosis and caspase-1/GSDMD-dependent pyroptosis in osteosarcoma cells. (A) Apoptosis rates measured by Annexin V-FITC/PI staining in LL-37-treated cells (n = 3). (B) TUNEL staining showing DNA fragmentation in osteosarcoma cells after LL-37 treatment. (C) Mitochondrial membrane potential (ΔΨm) detected by JC-1 staining (n = 3). (D) Intracellular ROS levels in osteosarcoma cells following LL-37 treatment (n = 3). (E) Quantitative analysis of JC-1 fluorescence intensity by flow cytometry (n = 3). (F) Western blotting of apoptosis-related proteins Bax, Bcl-2, cleaved caspase-3, and cleaved caspase-9. (G,H) Western blotting showing cleavage of caspase-1 and GSDMD, indicating pyroptosis activation. *p < 0.05, **p < 0.01, ***p < 0.001 vs. control group.

To elucidate the mechanisms underlying LL-37's anti-tumor effects, we first investigated its ability to induce cell death. Annexin V-FITC/PI staining by flow cytometry confirmed that LL-37 triggered apoptosis in both U2OS and MG63 cells in a concentration-dependent manner (Fig. 3A). This pro-apoptotic effect was further validated by TUNEL assay, which showed a dose-dependent increase in DNA fragmentation (Fig. 3B). Mechanistically, LL-37 treatment induced mitochondrial dysfunction, as evidenced by a concentration-dependent loss of mitochondrial membrane potential (ΔΨm) measured by both JC-1 fluorescence microscopy (Fig. 3C) and flow cytometry (Fig. 3E). Concurrently, intracellular reactive oxygen species (ROS) levels were substantially elevated upon LL-37 treatment (Fig. 3D). Western blot analysis demonstrated the activation of key apoptotic executors, confirming the involvement of the mitochondrial apoptosis pathway (Fig. 3F). Interestingly, LL-37 induced pyroptosis, marked by the cleavage of caspase-1 and gasdermin D (GSDMD), which drives this pro-inflammatory cell death and the release of damage-associated molecular patterns (DAMPs) and cytokines (e.g., IL-1β, IL-18) (Fig. 3G, H). Collectively, these findings demonstrate that LL-37 induces dual caspase-dependent cell death in OS cells, involving both mitochondrial apoptosis and pyroptosis.

### 3.4 Transcriptomic profiling identifies steroid biosynthesis as a key pathway modulated by LL-37 in osteosarcoma

**Fig. 4:**
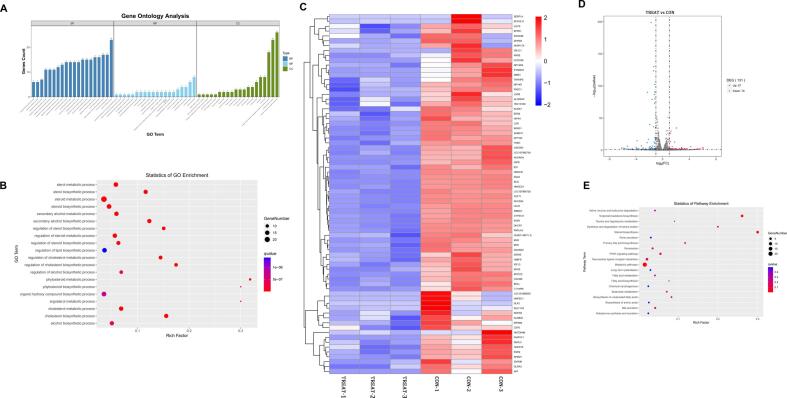
Transcriptomic profiling identifies steroid biosynthesis as the key pathway regulated by LL-37. (A) GO enrichment analysis of differentially expressed genes in U2OS cells. (B) KEGG pathway analysis showing steroid biosynthesis as the most significantly downregulated pathway. (C) Heatmap of differentially expressed genes related to cholesterol metabolism. (D) Volcano plot of global gene expression changes induced by LL-37. (E) Expression changes of core cholesterol synthesis genes including SQLE, HMGCR, and FDFT1.

To elucidate the molecular mechanisms underlying LL-37's action, we performed transcriptomic analysis on U2OS cells. RNA-sequencing revealed 131 differentially expressed genes (DEGs) upon LL-37 treatment, comprising 57 upregulated and 74 downregulated genes, as visualized in a volcano plot (Fig. 4D). Unsupervised clustering analysis clearly separated the transcriptomic profiles of LL-37-treated (TREAT) and control (CON) groups (Fig. 4C). Gene Ontology (GO) enrichment analysis of the DEGs demonstrated significant enrichment in biological processes and molecular functions related to steroid metabolism, detailed in both a summary bar chart and an enrichment scatter plot (Fig. 4A). Consistently, KEGG pathway analysis identified “Steroid biosynthesis” as one of the most significantly enriched pathways (Fig. 4B, E). Notably, key enzymes in the cholesterol biosynthetic pathway, including SQLE (squalene epoxidase), HMGCR (HMG-CoA reductase), and FDFT1, were among the most downregulated genes, with SQLE showing the most pronounced reduction. These transcriptomic findings suggest that disruption of cholesterol homeostasis may be a central mechanism underlying the anti-osteosarcoma activity of LL-37.

### 3.5 Elevated SQLE expression in osteosarcoma is linked to adverse prognosis and potential oncogenic pathways

**Fig. 5:**
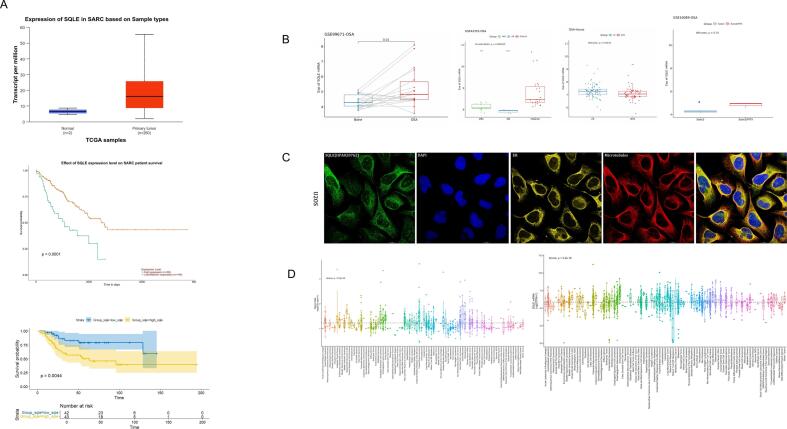
SQLE is highly expressed in osteosarcoma and predicts poor prognosis. (A) SQLE expression in osteosarcoma tissues vs. normal tissues in public databases. (B) Kaplan–Meier survival curve showing that high SQLE expression correlates with poor overall survival. (C) Immunofluorescence localization of SQLE in osteosarcoma cells. (D) SQLE DNA copy number gain and mRNA expression in osteosarcoma compared with other cancer types (CCLE dataset). *p < 0.05, **p < 0.01, ***p < 0.001 vs. normal or control group.

To assess the clinical and biological relevance of SQLE, a key gene downregulated by LL-37, we performed a multi-faceted analysis. Interrogation of public transcriptomic data confirmed that SQLEmRNA expression is significantly elevated in osteosarcoma tumor tissues compared to normal controls (Fig. 5A, top-left). Clinically, high SQLEexpression was strongly associated with significantly poorer overall survival in sarcoma patients (Figure A, middle-left), and risk stratification further validated its negative prognostic value (Fig. 5A, bottom-left). Independent dataset analysis corroborated the dysregulation of SQLE in osteosarcoma (Fig. 5B). Immunofluorescence staining showed that SQLE protein was predominantly localized intracellularly, specifically colocalizing with endoplasmic reticulum markers (Fig. 5C), aligning with its established role as a key enzyme in cholesterol biosynthesis. This genomic feature is strikingly prominent in osteosarcoma, as evidenced by analysis of the CCLE dataset (Fig. 5D, left subplot), which shows that osteosarcoma harbors the most significant SQLE DNA copy number gain among all profiled cancer types, significantly exceeding that of other bone cancers and most solid tumors (*p* < 0.0001). This amplification directly drives exceptionally high SQLE mRNA expression in osteosarcoma, placing it among the highest-expressing cancer types, with a strong positive correlation observed between copy number and expression levels (Fig. 5D, right subplot).In summary, the results of Fig.5D strongly support that SQLE can serve as a potential therapeutic target for OSA, especially for OSA patients with chemotherapy resistance or ineffective immunotherapy.

### 3.6 LL-37 suppresses cholesterol synthesis and induces apoptosis via the SREBP2/SQLE pathway in osteosarcoma

**Fig. 6:**
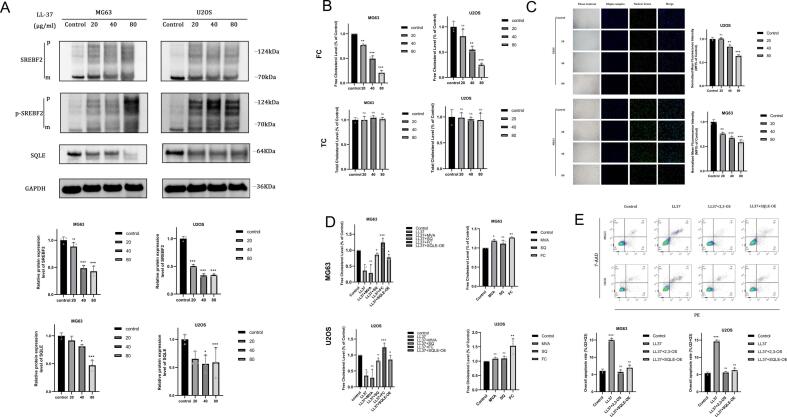
LL-37 suppresses cholesterol synthesis via the SREBP2/SQLE axis in osteosarcoma. (A) Western blotting showing downregulation of SREBP2 and SQLE by LL-37 in a dose-dependent manner. (B) Intracellular free cholesterol (FC) and total cholesterol (TC) levels after LL-37 treatment (n = 3). (C) Filipin III staining showing reduced free cholesterol in LL-37-treated cells. (D) Rescue of cholesterol levels by 2,3-oxidosqualene or SQLE overexpression (n = 3). (E) Apoptosis rescue assay confirming that SQLE mediates the pro-apoptotic effect of LL-37 (n = 3). *p < 0.05, **p < 0.01, ***p < 0.001 vs. control group; #p < 0.05 vs. LL-37 alone group.

Building on transcriptomic findings that cholesterol metabolism is a key target of LL-37, we further investigated its functional impact on the cholesterol biosynthesis pathway. Western blot analysis demonstrated that LL-37 treatment dose-dependently downregulated the key cholesterol synthesis transcription factor SREBP2 (both precursor and mature forms) and its critical downstream enzyme, SQLE, in MG63 and U2OS cells (Fig. 6A). Consistent with the suppression of this pathway, intracellular free cholesterol (FC) levels were significantly reduced upon LL-37 treatment, while total cholesterol (TC) levels showed no consistent change (Fig. 6B). This reduction in free cholesterol was visually confirmed by a decrease in Filipin complex staining intensity (Fig. 6C).

To establish a causal link between LL-37 and cholesterol synthesis inhibition, rescue experiments were performed. Supplementation with 2,3-oxidosqualene (2,3-OS), the metabolic product of SQLE, partially reversed the LL-37-induced reduction in free cholesterol. Conversely, overexpression of SQLE (SQLE-OE) effectively rescued the cholesterol-lowering effect of LL-37 (Fig. 6D). Functionally, the induction of apoptosis by LL-37, as measured by flow cytometry, was significantly attenuated by both 2,3-OS supplementation and SQLE overexpression (Fig. 6E). These data indicate that the pro-apoptotic effect of LL-37 is mediated, at least in part, through the inhibition of the SREBP2/SQLE axis and the subsequent reduction of intracellular cholesterol.

### 3.7 LL-37 downregulates SQLE via the PTEN/AKT/mTOR signaling pathway

**Fig. 7:**
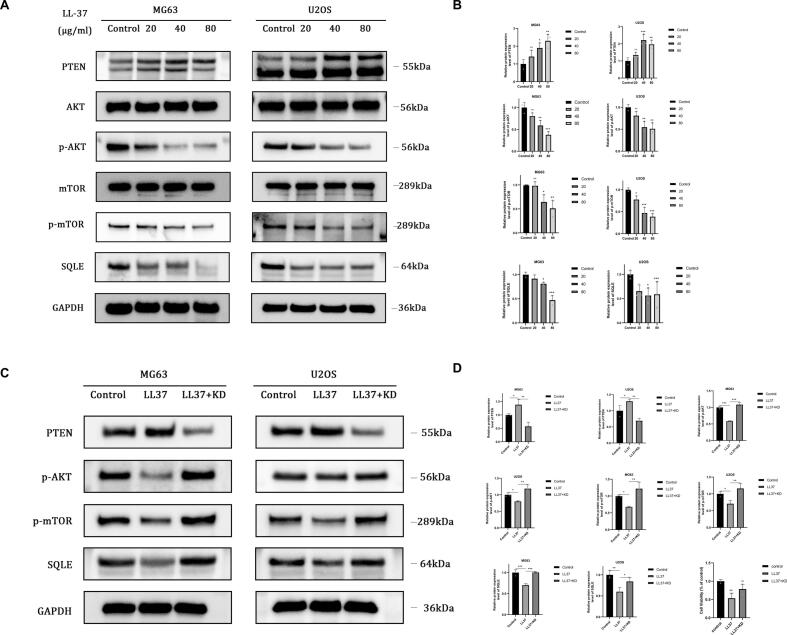
LL-37 downregulates SQLE through the PTEN/AKT/mTOR signaling pathway. (A,B) Western blotting showing upregulation of PTEN and inactivation of p-AKT and p-mTOR by LL-37. (C,D) PTEN knockdown abolishes the inhibitory effects of LL-37 on p-AKT, p-mTOR, and SQLE expression. *p < 0.05, **p < 0.01, ***p < 0.001 vs. control group; #p < 0.05 vs. LL-37 alone group.

To elucidate the upstream signaling mechanism underlying LL-37-mediated inhibition of SQLE expression, we examined the regulatory effect of LL-37 on the PTEN/AKT/mTOR pathway. Western blot assays revealed that LL-37 treatment dose-dependently elevated the protein expression of tumor suppressor PTEN in both MG63 and U2OS osteosarcoma cell lines (Fig. 7A). Correspondingly, LL-37 suppressed the phosphorylation (a marker of protein activation) of key signaling molecules AKT and mTOR, whereas the total protein levels of these two kinases remained unaltered (Fig. 7A, B). This suppression of the AKT/mTOR pathway was concurrently associated with a significant downregulation of SQLE protein expression (Fig. 7A, B).

To confirm the causal involvement of PTEN in this regulatory cascade, we conducted a loss-of-function assay via PTEN-specific small interfering RNA (siRNA) transfection. PTEN knockdown effectively abrogated the LL-37-induced suppression of phosphorylated AKT (p-AKT), phosphorylated mTOR (p-mTOR), and SQLE in both cell lines (Fig. 7C, D). These findings indicate that PTEN acts as a critical upstream mediator: LL-37 downregulates SQLE in osteosarcoma cells by activating PTEN, which in turn suppresses the AKT/mTOR signaling axis.

### 3.8 LL-37 effectively inhibits tumor growth in an osteosarcoma xenograft model with a favorable safety profile

**Fig. 8:**
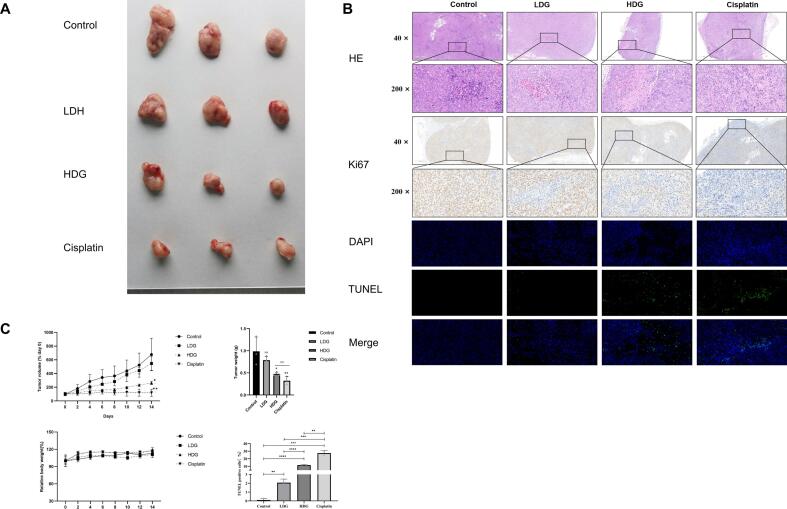
LL-37 inhibits osteosarcoma xenograft growth in vivo with favorable safety. (A) Representative images of excised tumors from control, LL-37 low-dose, high-dose, and cisplatin groups. (B) H&E, Ki-67 IHC, and TUNEL staining of tumor tissues from each group. (C) Tumor volume growth curve, final tumor weight, and mouse body weight changes (n = 3 mice per group). *p < 0.05, **p < 0.01, ***p < 0.001 vs. control group.

To evaluate the in vivo anti-tumor efficacy of LL-37, we established a xenograft mouse model using 143B OS cells. Macroscopic assessment of excised tumors revealed a clear, dose-dependent reduction in tumor size in the low-dose (LDG) and high-dose (HDG) LL-37 treatment groups compared to the control group, with the HDG effect being comparable to the cisplatin-positive control (Fig. 8A). Histological analysis via H&E staining showed that tumors from the control group exhibited high cellular density and disorganized architecture.In contrast, tumors from the LDG, HDG, and cisplatin groups exhibited reduced cellularity and a looser tissue architecture (Fig. 8B, H&E panels). Furthermore, immunohistochemical staining for the proliferation marker Ki67 revealed a marked reduction in Ki67-positive cells in the tumors of all treatment groups, with the most pronounced decrease observed in the HDG and cisplatin groups, indicative of robust suppression of tumor cell proliferation (Fig. 8B, Ki67 panels). Additionally, TUNEL fluorescent staining demonstrated a notable increase in TUNEL-positive cells in the high-dose LL-37 group, reflecting enhanced apoptotic cell death in the tumor tissue (Fig. 8B, TUNEL panels).

Quantitative measurements corroborated these observations. The growth curves of tumor volume over time showed that both LDG and HDG LL-37 treatments significantly slowed tumor growth compared to the control, with HDG achieving near-complete suppression similar to cisplatin (Fig. 8C, top-left). At the experimental endpoint, the tumor weights in the HDG and cisplatin groups were significantly lower than that of the control group (Fig. 8C, top-right). Crucially, no significant body weight loss was observed in mice treated with either dose of LL-37 throughout the study period, indicating a favorable safety profile and minimal systemic toxicity (Fig. 8C, bottom-left). Collectively, these in vivo results demonstrate that LL-37 potently inhibits osteosarcoma tumor growth and proliferation with efficacy comparable to standard chemotherapy, while exhibiting a favorable safety window.

## 4 Discussion

Osteosarcoma (OS) remains a clinically intractable primary malignant bone tumor [1], with stagnant 5-year survival rates and dismal prognoses for metastatic/recurrent disease [5], driven by its genomic heterogeneity, chemoresistance, and immunosuppressive microenvironment [8]. The urgent need for novel, selective, and safe therapeutic agents has prompted exploration of unconventional targets, including antimicrobial peptides and metabolic reprogramming [34], [35]. In this study, we identify the human cathelicidin LL-37 as a potent anti-OS agent and elucidate its novel molecular mechanism: LL-37 upregulates the tumor suppressor PTEN to inhibit the AKT/mTOR signaling axis, thereby suppressing the SREBP2/SQLE-mediated cholesterol biosynthesis pathway, and concurrently induces mitochondrial apoptosis and caspase-1/GSDMD-dependent pyroptosis in OS cells. These effects are selective for OS cells, with minimal cytotoxicity to normal human bone marrow mesenchymal stem cells (hBMSCs), and translate to robust in vivo tumor growth inhibition with a favorable safety profile, establishing LL-37 as a promising therapeutic candidate for OS and uncovering a novel PTEN/AKT/mTOR-SREBP2/SQLE regulatory axis in OS pathogenesis.

A defining advantage of LL-37 as an anti-OS agent is its tumor cell selectivity, a critical attribute for cancer therapeutics given the severe systemic toxicity of standard OS chemotherapy (cisplatin, doxorubicin, methotrexate). Our in vitro assays demonstrate that LL-37 potently inhibits the viability of U2OS and MG63 OS cells (IC50 values of 54.01 μg/mL and 62.77 μg/mL at 24 h, respectively) but exerts negligible effects on hBMSCs even at concentrations up to 640 μg/mL—10-fold higher than the OS cell IC50(Fig. 1). We further evaluated the hemolytic activity of LL-37 using whole blood collected from BALB/c nude mice, which serves as a key indicator for its biosafety. After 24 h incubation at 37 °C, the hemolysis rate remained below 5% across all tested LL-37 concentrations ranging from 20 to 80 μg/mL. Consistent with published findings, LL-37 exhibits mild hemolytic potency against serum-free purified erythrocytes at concentrations above 50–100 μg/mL. Ciornei et al. reported that approximately 89.9 μg/mL LL-37 induced 5.8% hemolysis in purified human red blood cells after short-term incubation [36]. Another study revealed that even at the extremely high concentration of 175 μg/mL, LL-37 only caused around 4.47% hemolysis in purified erythrocytes [37]. Furthermore, the hemolytic capacity of LL-37 is strongly pH-dependent: it hardly damages erythrocyte membranes under physiological neutral conditions, whereas prominent hemolysis is only detected in acidic environments [38]. The difference in hemolytic performance between purified erythrocyte preparations and whole blood is mainly attributed to plasma components. Albumin and apolipoprotein A-I in native blood can bind to cationic LL-37, shield its membrane-disrupting domain and markedly attenuate its hemolytic activity. In vivo data further verify the favorable safety profile of LL-37: intravenous administration of LL-37 at the maximum dose of 16 mg/kg in mice resulted in a hemolysis rate lower than 0.5%, with no elevation of plasma free hemoglobin in either infected or uninfected animals [39]. Collectively, the working concentrations of LL-37 adopted in this study possess excellent biosafety and are suitable for subsequent in vivo application.

This selectivity is consistent with emerging reports of context-dependent LL-37 activity in cancer, where its anti-tumor effects are restricted to specific malignancies (e.g., colorectal cancer, glioblastoma) [40] [41] while exhibiting pro-tumor activity in others (e.g., breast, lung cancer) [42], [43]. For OS, this selectivity may stem from the unique molecular and metabolic features of OS cells, including dysregulated PTEN/AKT/mTOR signaling [44] [45] and hyperactivated cholesterol biosynthesis [32], [46]—pathways we identify as key LL-37 targets—whereas normal hBMSCs maintain homeostatic regulation of these axes [47], [48], rendering them insensitive to LL-37-mediated modulation. This selectivity not only mitigates the risk of off-target bone marrow toxicity (a major limitation of standard OS chemotherapy) but also supports LL-37 as a candidate for localized or systemic delivery with a wide therapeutic window.

Our functional assays confirm that LL-37 exerts comprehensive anti-OS effects by abrogating core oncogenic properties: it suppresses long-term clonogenic survival, impairs migratory and invasive capacity, and induces S-phase cell cycle arrest in OS cells (Fig. 2). These phenotypic changes are underpinned by dual induction of programmed cell death [49]—mitochondrial apoptosis and pyroptosis—a novel finding for LL-37 in OS and a critical mechanism for overcoming cancer cell resistance to single-mode cell death induction(Fig. 3). LL-37 triggers mitochondrial dysfunction, as evidenced by reduced mitochondrial membrane potential (ΔΨₘ) and elevated intracellular reactive oxygen species (ROS), which activates the intrinsic apoptotic pathway (upregulated Bax, downregulated Bcl-2, and cleaved caspase-3/9)(Fig. 3C-F). Concurrently, LL-37 induces pyroptosis via cleavage of caspase-1 and GSDMD, a pro-inflammatory form of cell death that releases damage-associated molecular patterns (DAMPs) and pro-inflammatory cytokines (e.g., IL-1β, IL-18)(Fig. 3G,H). In the immunosuppressive OS microenvironment, pyroptosis may exert additional anti-tumor effects by recruiting and activating immune cells, potentially reversing immune tolerance [50]—an effect that warrants further investigation in immune-competent OS models. The induction of dual cell death programs makes LL-37 particularly attractive for OS, where resistance to apoptosis is a major driver of chemoresistance and treatment failure [51].

Transcriptomic profiling was a pivotal step in identifying cholesterol biosynthesis as a central LL-37 target in OS, with KEGG pathway analysis highlighting “Steroid biosynthesis” as the most significantly enriched downregulated pathway (Fig. 4B,E). Among the key cholesterol biosynthetic enzymes suppressed by LL-37, SQLE—a rate-limiting enzyme catalyzing the conversion of squalene to 2,3-oxidosqualene—exhibited the most pronounced downregulation(Fig. 4C,D), and our clinical relevance analysis confirmed that SQLE is a bona fide oncogene in OS (Fig. 5A,B). SQLE mRNA and protein are significantly upregulated in OS tumor tissues compared to normal controls, and high SQLE expression is strongly associated with poor overall survival in sarcoma patients. Notably, OS harbors the most significant SQLE DNA copy number gain among all profiled cancer types (CCLE dataset), which directly drives exceptionally high SQLE expression—an amplicon that is rare in normal bone tissue and other bone cancers(Fig. 5D). These findings align with previous reports of SQLE upregulation in OS and its association with adverse clinical outcomes [27], [28], [52], [53], and extend this work by establishing SQLE as a downstream target of LL-37 and a critical mediator of OS cell survival via cholesterol homeostasis. Rescue experiments further confirm the causal role of SQLE inhibition in LL-37's anti-OS effects: supplementation with 2,3-oxidosqualene (the SQLE metabolic product) or SQLE overexpression partially reverses LL-37-induced reductions in intracellular free cholesterol and abrogates LL-37-mediated apoptosis(Fig. 6B-E), validating that SREBP2/SQLE suppression is a necessary component of LL-37's mechanism of action.

Cholesterol biosynthesis is tightly regulated by the SREBP2 transcription factor, whose activation is known to be modulated by the PI3K/AKT/mTOR signaling axis—a pathway frequently dysregulated in OS via loss of PTEN function or AKT/mTOR amplification [54], [55], [56]. Our study uncovers a novel PTEN/AKT/mTOR-SREBP2/SQLE regulatory cascade in OS and identifies LL-37 as a modulator of this axis. We demonstrate that LL-37 dose-dependently upregulates PTEN protein expression in OS cells, which in turn suppresses the phosphorylation (activation) of AKT and mTOR (without altering total AKT/mTOR levels)(Fig. 7A). PTEN knockdown abrogates LL-37-mediated suppression of p-AKT, p-mTOR, and SQLE, confirming PTEN as the critical upstream mediator of LL-37's effects on the AKT/mTOR-SREBP2/SQLE axis(Fig. 7B). This finding is significant for two reasons: first, it identifies a novel mechanism of PTEN-mediated regulation of cholesterol biosynthesis in OS, linking the classical tumor suppressor function of PTEN to metabolic reprogramming [57], [58]—a burgeoning area of cancer research; second, it explains why LL-37 exerts selective effects on OS cells, which frequently exhibit PTEN loss or downregulation [59], [60], [61], whereas normal hBMSCs maintain functional PTEN [62], [63] and thus do not require LL-37-mediated PTEN upregulation for homeostatic signaling. OS patients are notably conserved, as canine osteosarcoma likewise [64] exhibits frequent PTEN deficiency or AKT/mTOR pathway amplification, mirroring the poor-prognosis subgroups observed in human OS patients [65], [66]. LL-37 may represent a targeted therapy to reactivate PTEN and suppress hyperactivated cholesterol biosynthesis, a strategy that is not currently addressed by standard OS treatments.

Our in vivo xenograft studies confirm the translational potential of LL-37, demonstrating that intraperitoneal administration of LL-37 (5 mg/kg and 10 mg/kg every other day for 2 weeks) induces a dose-dependent reduction in 143B OS tumor growth, with the high-dose LL-37 group exhibiting efficacy comparable to cisplatin (5 mg/kg)—the gold-standard OS chemotherapy(Fig. 8A,C). Histological, immunohistochemical, and TUNEL fluorescence analyses collectively demonstrated that LL-37 suppresses tumor cell proliferation (evidenced by reduced Ki67-positive cells) and promotes cell death (indicated by increased TUNEL-positive signals) in vivo(Fig. 8C), consistent with the observations from in vitro experiments. Most importantly, LL-37 treatment is associated with no significant body weight loss in mice(Fig. 8C), whereas cisplatin (a known nephrotoxic and myelosuppressive agent) typically induces weight loss in preclinical models [67], [68]—an observation that supports the favorable safety profile of LL-37 and its potential to avoid the severe systemic toxicity of standard OS chemotherapy. This in vivo efficacy, combined with its in vitro selectivity, positions LL-37 as a promising candidate for further preclinical and clinical development, either as a monotherapy or in combination with standard chemotherapy to enhance efficacy and reduce toxicity.

This study has several limitations that warrant further investigation. First, all in vivo experiments were performed using immunocompromised BALB/c nude mice, which lack mature T cells and a functional adaptive immune system [69], [70]. We chose this model to eliminate the interference of host adaptive immunity, allowing us to specifically characterize the direct anti-tumor effects of LL-37 and its regulation of cholesterol metabolism, the core focus of this research. However, this immunodeficient strain cannot recapitulate the complete tumor immune microenvironment, restricting the extrapolation of our findings to immunocompetent animals and clinical settings. Since LL-37 induces pyroptosis and exerts immunomodulatory activities, future studies will adopt syngeneic OS models [71], [72] and patient-derived xenograft (PDX) models to explore the crosstalk between LL-37-triggered cell death and anti-tumor immunity, and further validate its translational potential as an immunotherapeutic adjuvant.

Second, a notable discrepancy exists between LL-37 concentrations used for in vitro cell culture and in vivo administration. It should be emphasized that the relatively low in vivo dosages (5 mg/kg and 10 mg/kg) were not limited by systemic toxicity. As a cationic peptide, LL-37 can bind to albumin and apolipoprotein A-I in plasma, which masks its membrane-active domain and reduces the level of free active peptide in circulation [39]. A published preclinical study adopting the same intraperitoneal administration and mouse xenograft model reported that 20 mg/kg LL-37 exerted obvious anti-tumor efficacy without evident systemic toxicity [73]. Pharmacologically, in vitro culture requires high local concentrations for direct cell intervention, while systemic administration enables LL-37 to circulate and accumulate in tumor tissues, so low doses can achieve satisfactory efficacy. Referencing this evidence, we selected 5 mg/kg as the low dose and 10 mg/kg as the maximum well-tolerated dose in vivo administration. Although our highest in vivo dosage was lower than the 20 mg/kg dose described in the literature, LL-37 still produced robust anti-osteosarcoma effects in xenograft models. To improve LL-37 stability, tumor targeting, bioavailability and in vivo efficacy for clinical translation, novel formulations including modified peptides, nanoparticles and liposomes need to be developed in follow-up work [74].In addition, only three BALB/c nude mice were used per xenograft group in the present study. This sample size is relatively limited for anti-tumor efficacy assessment, and we plan to expand animal numbers to *n* ≥ 6 in subsequent independent animal experiments to minimize individual variation and validate our findings more robustly.

Third, this study mainly focused on the PTEN/AKT/mTOR-SREBP2/SQLE axis and dual cell death pathways induced by LL-37. As a pleiotropic peptide, LL-37 also interacts with other oncogenic molecules such as EGFR and MAPK [75], [76]. Future research should clarify whether these signaling pathways participate in the anti-OS actions of LL-37, as well as its regulatory roles in glycolysis, fatty acid synthesis and other metabolic networks.

Fourth, we did not evaluate the efficacy of LL-37 in chemoresistant OS models, which remains a major unmet clinical need [77].Further experiments using cisplatin- or doxorubicin-resistant OS cell lines and xenografts are required to determine whether LL-37 can overcome chemotherapy resistance.

Finally, our analysis of the clinical relevance of SQLE was based on public transcriptomic datasets. Subsequent validation in a large cohort of primary OS samples with complete clinical follow-up data is necessary. Meanwhile, we will explore the correlation between PTEN/SQLE expression levels and cellular sensitivity to LL-37 using patient-derived OS models.

## AI writing declaration

No generative artificial intelligence tools were used for manuscript drafting, data analysis, image generation or experimental design in this work.

## Copyright & originality declaration

The manuscript is original research completed by all listed authors. This paper has not been published previously and is not under consideration for publication in any other journal. All authors consent to the submission of this manuscript to Journal of Bone Oncology and confirm that all content complies with the journal's publication ethics and copyright regulations.

## CRediT authorship contribution statement

**Fatai Lu:** Writing – original draft, Resources, Project administration, Methodology, Investigation, Data curation, Conceptualization. **Zhijun Liu:** Software. **Huasheng Jiang:** Investigation. **Hong Zheng:** Data curation. **Wenhui Su:** Project administration. **Baosen Zhou:** Writing – review & editing, Supervision, Resources.

## Clinical trial number

Not applicable.

## Consent for publication

This manuscript does not contain any individual person's data in any form (including individual details, images, or videos). Not applicable.

## Ethics approval and consent to participate

All animal experiments were approved by the Institutional Animal Care and Use Committee of China Medical University (Approval No.: CMU20231310; Approval Date: 2023-11-15). The study was conducted in compliance with the SYXK (Liao) 2022–0007 (the Laboratory Animal Use License of China Medical University) and the National Institutes of Health Guide for the Care and Use of Laboratory Animals (NIH Publications No. 8023, revised 1978). All experimental procedures strictly followed the approved animal welfare and ethical review protocols for this project. This study did not involve human subjects or human biological samples, so informed consent for participation is not applicable.

## Funding

This work was supported by the 10.13039/501100005047Natural Science Foundation of Liaoning Province (No. 2023-MS-166). The funding body had no role in the design of the study, collection, analysis and interpretation of data, writing of the manuscript, or decision to submit the article for publication.

## Declaration of competing interest

The authors declare that they have no known competing financial interests or personal relationships that could have appeared to influence the work reported in this paper.
